# External Morphology, Defensive Adaptations, Aposematic Coloration, and Sexual Dimorphism of the Fifth Instar Larva of Cricula Silkmoth, *Cricula trifenestrata* Helfer (Lepidoptera: Saturniidae) from Thailand

**DOI:** 10.3390/insects16020105

**Published:** 2025-01-21

**Authors:** Kanitsara Magnussen, Motoyuki Sumida, Anongrit Kangrang, Fritz Vollrath, Teeraporn Katisart, Chirapha Butiman

**Affiliations:** 1 Department of Biology, Faculty of Science, Mahasarakham University, Kantharawichai District, Maha Sarakham 44150, Thailand; teeraporn@msu.ac.th; 2 The Research Unit of Center of Excellence for Mulberry and Silk, Center of Excellence for Silk Innovation, Mahasarakham University, Kantharawichai District, Maha Sarakham 44150, Thailand; chirapha_b@msu.ac.th; 3 Center of Excellence for Silk Innovation, Mahasarakham University, Kantharawichai District, Maha Sarakham 44150, Thailand; mtysumida@gmail.com; 4 Department of Environmental Engineering, Faculty of Engineering, Mahasarakham University, Kantarawichai District, Maha Sarakham 44150, Thailand; anongrit.k@msu.ac.th; 5 Department of Zoology, University of Oxford, Oxford OX1 3PS, UK; fritz.vollrath@biology.ox.ac.uk; 6 Upper Woods Farm, Beckley, Oxford OX3 9TF, UK

**Keywords:** aposematic coloration, *Cricula trifenestrata* Helfer, fifth instar larva, fluorescence hair warts, sex dimorphism, scoli

## Abstract

This study explores the external morphology of larva of *Cricula trifenestrata* Helfer at the fifth instar stage, focusing on sexual dimorphism, scoli, and fluorescence hair warts. The larva displays a black body adorned with varying shades of orange to crimson–red transverse stripes and small yellow dorsal spots. Longitudinal stripes with fluorescent warts are observed in the subspiracular region, accompanied by an overall coverage of long white hairs. These distinctive features, including scoli and fluorescence hair warts, serve as effective defense mechanisms against predators and parasitoids. The results enhance our understanding of *C. trifenestrata* Helfer larval biology, providing valuable insights for entomology and evolutionary biology. The identification of species-specific adaptations, particularly the presence of scoli and fluorescence hair warts, underscores their significance in shaping survival strategies and ecological interactions.

## Simple Summary

The *Cricula trifenestrata* Helfer, a wild silkmoth found in various Southeast Asian countries, is often considered a pest due to the damage its larvae cause to crops. However, its silk, which is more valuable than that of the common silkworm, holds significant economic potential. This study focuses on examining the larvae found on cinnamon trees in Thailand in order to gain a better understanding of their physical characteristics. The larvae have distinctive black and crimson–red bodies with striking yellow spots and long whitish hairs, which may serve as a defense mechanism against predators. The researchers also discovered a unique luminescent quality in the yellow hair warts under regular and UV light, indicating a possible role in defense. Furthermore, the authors observed differences between male and female larvae, with females being larger and having different stripe patterns. This study offers a detailed description of the larvae’s morphology, providing valuable insights that could aid in managing their impact on crops and maximizing the potential benefits they offer. Understanding these characteristics could lead to more effective pest control methods and enhanced utilization of their silk, thus making a positive contribution to agriculture and the silk industry.

## 1. Introduction

Cricula Silkmoth, *Cricula trifenestrata* Helfer (Lepidoptera: Saturniidae), is known as a pest causing severe crop damage in countries like India, Sri Lanka, Bangladesh, Myanmar, Malaysia, Vietnam, Philippines, Indonesia, Singapore, Laos, Cambodia, and Thailand [1,2,3]. The silk fiber from their cocoons is, however, highly valued, surpassing *Bombyx* silk fiber [4]. Potential health benefits from the silk powder are reported, including cholesterol control and antioxidant activity [1]. The pupae serve as a nutritious food source [5]. Indonesia initiated a large-scale culture of Cricula silkmoths, drawing interest from India and Thailand, because insect pests were transformed to value-added products [2,3].

We reported the presence of *C. trifenestrata* Helfer on Cinnamon trees (*Cinnamomum* sp.) in an orchard in Chaiyaphum province, Northeastern Thailand, and described the general features of the fifth instar larvae, pupae, moths, and cocoons, as well as taxonomic characters such as male wing venation and genitalia [3].

On the larval morphology of *C. trifenestrata* Helfer, Hampson (1892) reported that larvae are blackish–brown and bear six setiferous tubercles on the 2nd to 11th somites [6]. Rono (2008) characterized fully grown fifth instar larvae as elongated and cylindrical, symmetrically arranged with transverse bands of black, yellow, and red on the dorsal part of each segment [7]. Tikader (2014) recorded that the fifth instar larval body is dark brown to orange, with pinkish bands and yellow spots on the dorsal surface, and alternating bands of black, yellow, and red on the thorax and abdomen [1]. We identified the fifth instar larva as black with transverse crimson–red strips decorated with spine-like setae and pink tubercles (scoli) extending long whitish hair. The dorsal part exhibited numerous glowing yellow dots and stripes. Two distinct stripe patterns were also observed [3].

However, there is a lack of detailed description on the external morphology of larval body parts as the head, thorax, and abdomen, as well as the structure of scoli and yellow hair warts. Two distinct stripe patterns of *C. trifenestrata* Helfer [3] were reinvestigated and discussed, related to sexual dimorphism [8].

## 2. Materials and Methods

### 2.1. Collection of Larvae

**Figure 1:**
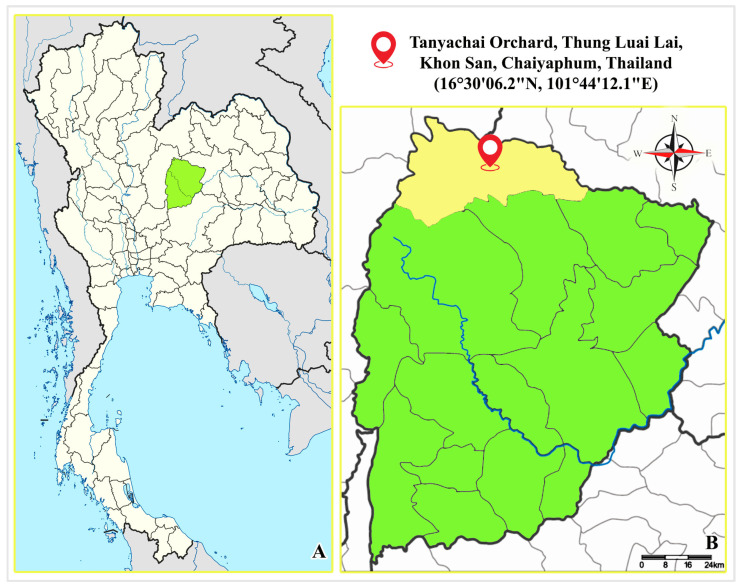
Location of Tanyachai Orchard in Chaiyaphum Province, Thailand. (**A**) Map of Thailand with Chaiyaphum Province highlighted in green (adapted from Wikimedia Commons [14]). (**B**) Enlarged map of Chaiyaphum Province showing Khon San District highlighted in yellow, with Tanyachai Orchard marked by a red pin in Thung Luai Lai Subdistrict (adapted from Wikimedia Commons [15]).

In August 2023, 20 fifth instar larvae of *Cricula trifenestrata* Helfer were collected from the Tanyachai Orchard, located in Thung Luai Lai Subdistrict, Khon San District, Chaiyaphum Province, Thailand (coordinates: 16°30′06.2″ N, 101°44′12.1″ E; see map in Figure 1). The larvae were found on cinnamon leaves and carefully secured in blue nylon net bags along with foliage to ensure their safety during transportation.

### 2.2. General Observation

The living larvae were transported to the laboratory at the Department of Biology, Faculty of Science, Mahasarakham University, for further examination. The larvae were measured and photographed using a modified Nikon camera lens. Some larvae were preserved by freezing at −20 °C until further use, while others were documented under a stereo microscope.

### 2.3. Description of Morphology

The morphological description of the larvae was conducted utilizing the established terminologies outlined by Deml and Dettner (2002) [9], Headrick and Gordh (2009) [10], Poletto et al. (2010) [11], and Miller and Hammond (2013) [12]. Finally, the sex of the larvae was determined using the method described by Aruga (1994) [13].

## 3. Results

### 3.1. External Morphology of the Fifth Instar Larva

#### 3.1.1. General Morphology

**Figure 2:**
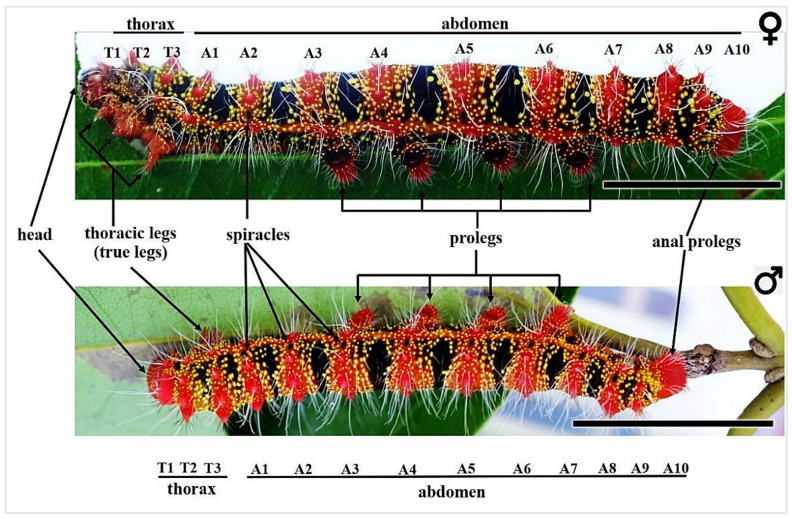
A fifth instar larvae of both sexes of *C. trifenestrata* Helfer, with their anatomical regions such as the head, thorax (T1–T3), and abdomen (A1–A10) are shown. The figure also shows the presence of thoracic legs, abdominal prolegs, anal prolegs, and spiracles (scale bar = 2 cm).

The larva of the fifth instar of *C. trifenestrata* Helfer is characterized by a black body with transverse bands that range in color from orange to crimson–red. It is adorned with small yellow spots present around the dorsal part of its body and in the lateral longitudinal stripe (Figure 2). The body of the larva is covered with long whitish hairs. In the fifth instar stage, the larva of *C. trifenestrata* Helfer exhibits a long, cylindrical body that consists of three distinct segments—the head, thorax, and abdomen—as illustrated in Figure 2. The fifth instar *C. trifenestrata* Helfer larva specimens measure approximately 6.5–7.5 cm in length and 0.85–1 cm in width, and are divided into three main segments: the head (0.5 cm long, 0.5 cm wide), thorax (1 cm long, 0.85–1 cm wide), and abdomen (5.5–6 cm long, 0.85–1 cm wide).

A detailed description of each part reveals the specific characteristics of the larva as follows:

#### 3.1.2. Head

The head is the anterior part of the larva, consisting of the head capsule, which has additional organs such as the stemmata, mouth, and antennae.

**Figure 3:**
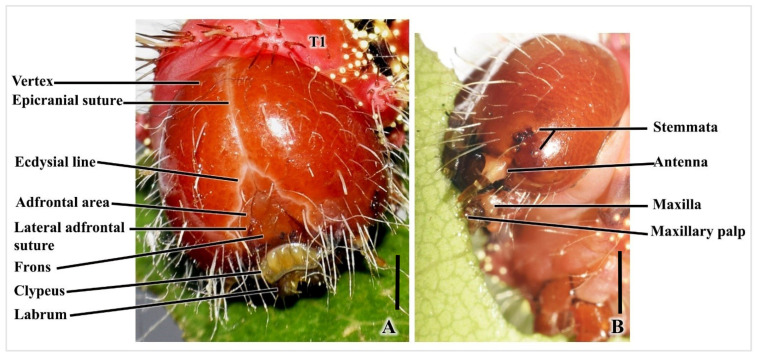
Fifth instar larva of *C. trifenestrata* Helfer head. (**A**) Frontal view. (**B**) Lateral view, with important components labeled (scale bar = 1 mm).

**Figure 4:**
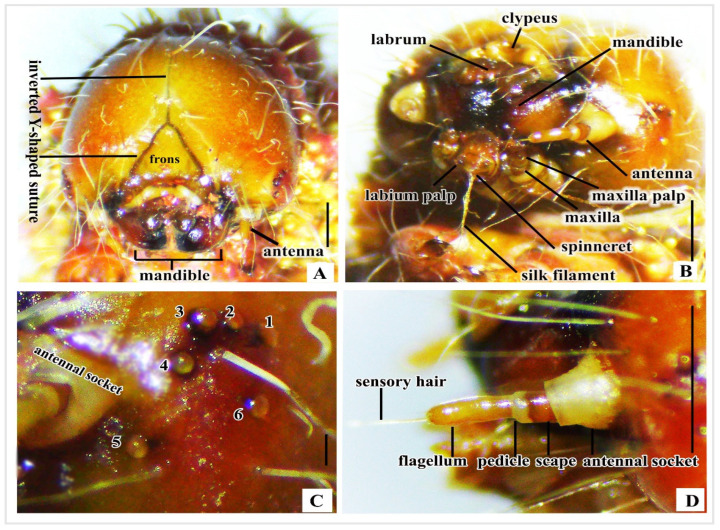
Components of *C. trifenestrata* Helfer larva’s head: (**A**) Frontal view. (**B**) Mouthpart components. (**C**) Stemmata location, with numbers 1–6 representing individual stemmata. (**D**) Antennae components (scale bar (**A**–**D**) = 1 mm).

Head capsule: This is characterized by a spherical, crimson–red, sclerotized, hypognathous orientation. Two distinct lobes are connected by an inverted Y-shaped suture (Figure 3A and Figure 4A). Epicranial suture: A coronal suture initiates from the top of the epicranial notch, extending down to join an inverted V-shaped lateral adfrontal suture (Figure 3A and Figure 4A). Edysial lines: White lines above the adfrontal suture are more distinct in live larvae. The adfrontal area is between the edysial line and adfrontal suture. Frons: The triangular frons is enclosed within the lateral adfrontal suture (Figure 3A and Figure 4A).

Mouthparts: There is a clypeus with a bean-shaped reniform surface, as well as five lobes in white, cream, or yellow, bordered by white edges (Figure 3A and Figure 4B). The labrum has a central notch and short setae (Figure 3A and Figure 4B). Robust mandibles are adorned with black–brown tips (Figure 4A,B). The labium is centrally positioned between two maxillae. An anterior labium section with a pair of labial palps and a median spinneret are for silk filament secretion. The maxillary teeth terminus contains maxillary pulp (Figure 4B).

Stemmata: Simple eyes are arranged semi-circularly around the gena (stemmata 1–4, 6), with a fifth near the base of the antenna (Figure 4B,C).

Antennae: These are composed of four segments: a base inside the white antenna socket, a deep brown scape, an off-white pedicle, and a long light brown flagellum. Sensory hair extends from the end of the flagellum (Figure 3B and Figure 4D).

#### 3.1.3. Thorax Segments

**Figure 5:**
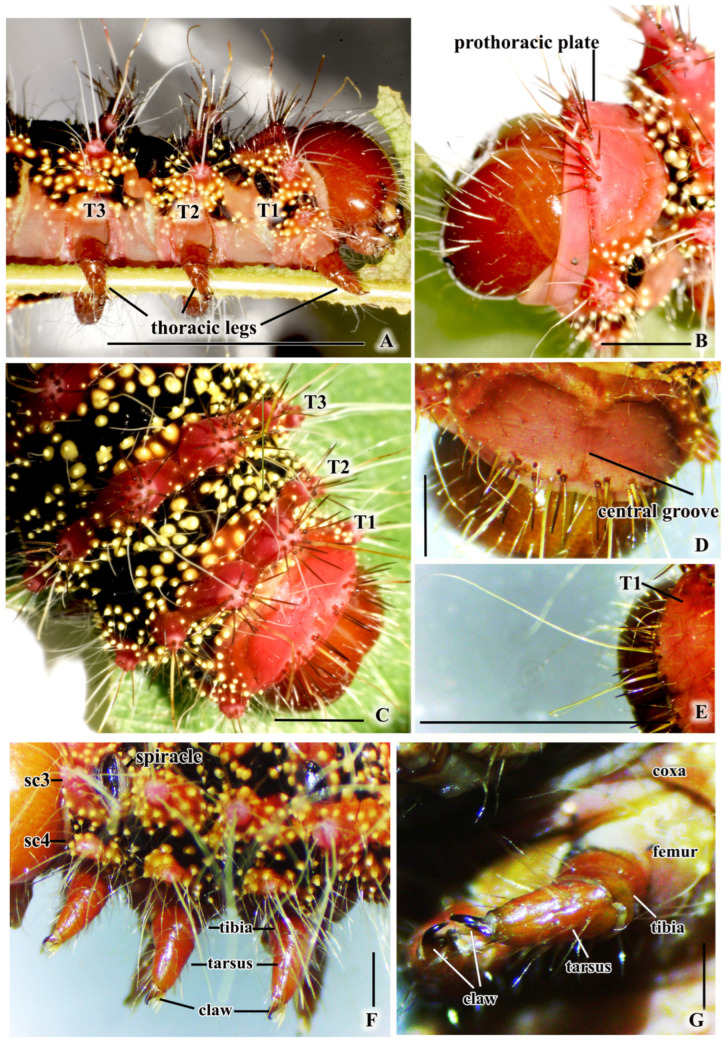
Components of *C. trifenestrata* Helfer larva’s thorax. (**A**) The lateral view of the thorax segment (T1–T3) (scale bar = 1 cm). (**B**) The prothoracic plate (scale bar = 3 mm). (**C**) Thorax scoli (scale bar = 3 mm). (**D**) The central groove on a prothoracic plate (scale bar = 3 mm). (**E**) The whitish hair of scoli on a thoracic plate (scale bar = 6 mm). (**F**) Scoli (Sc3 and Sc4), spiracle, and thoracic legs on thorax segment (scale bar = 1 mm). (**G**) The structure of the prothoracic leg, which includes coxa, femur, tibia, tarsus, and claw (scale bar = 1 mm).

The thorax of the larva can be divided into three segments: prothorax (T1), mesothorax (T2), and metathorax (T3) (Figure 2 and Figure 5A). Each of these segments has a pair of true legs that are brown–red and sclerotized, with four jointed parts consisting of a coxa, femur, tibia, and tarsus. The end of the tarsus is dark and has a hooked claw (Figure 5A,F,G).

**Figure 6:**
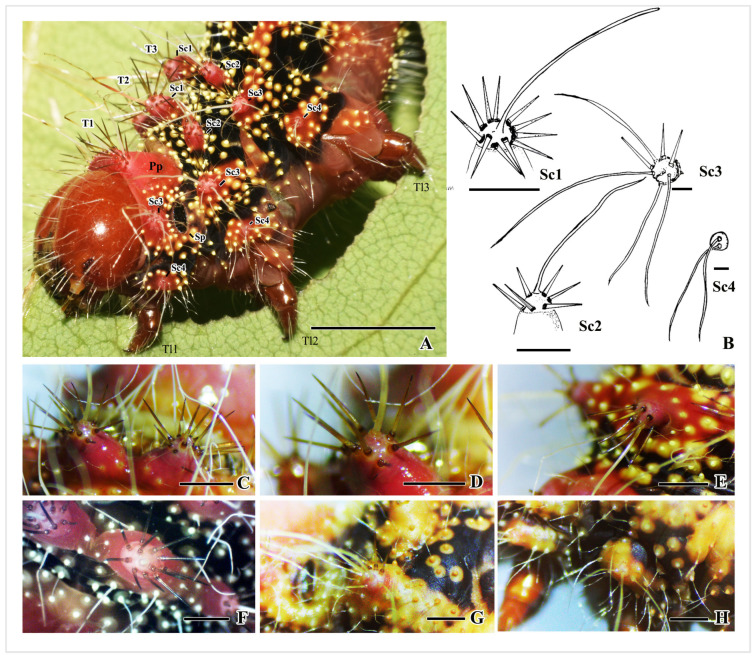
Scoli on the thorax segments of *C. trifesnestrata* Helfer. (**A**) The location of the four types of scoli on the thorax (scale bar = 1 cm). (**B**) Four types of scoli of the thorax: Sc1 (scale bar = 923 µm); Sc2 (scale bar = 640 µm); Sc3 (scale bar = 350 µm); Sc4 (scale bar = 150 µm). (**C**) Pair of Sc1 on the T2 segment. (**D**) Sc1. (**E**) Sc2. (**F**) Sc1 on T3. (**G**) Sc3 on T2. (**H**) Sc4 on T2 (scale bar (**C**–**H**) = 1 mm).

**Figure 7:**
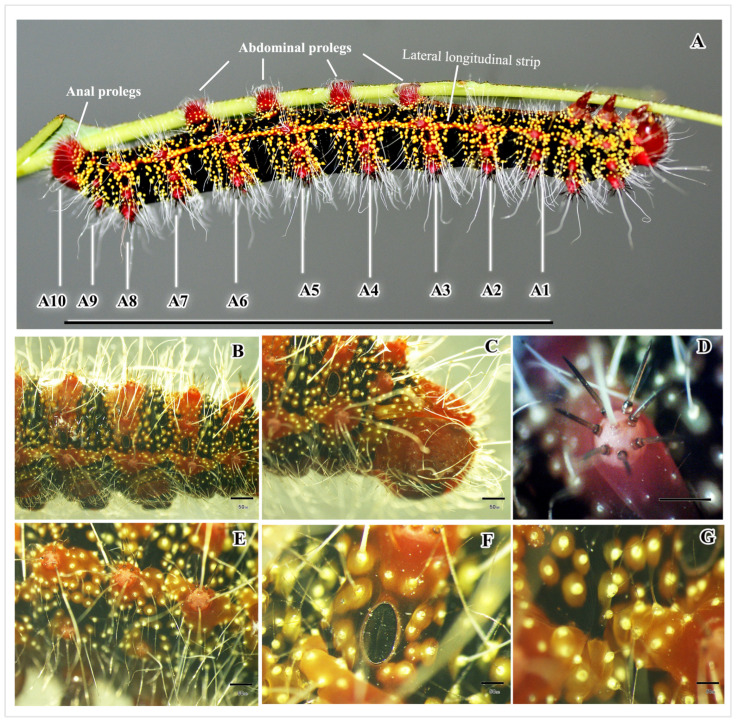
The abdominal segments of *C. trifenestrata* Helfer larva. (**A**) The lateral view of the body is labeled as the abdominal segments A1–10, anal plate, abdominal prolegs, and lateral longitudinal strip (scale bar = 5.5 cm). (**B**) The side view of segments A4–8 shows the fluorescence of the hair warts (scale bar = 50 µm). (**C**) Segments A8–10 show the anal plate (scale bar = 50 µm). (**D**) The scoli at the dorsal part includes 7 setae and 1 long white hair (scale bar = 50 µm). (**E**) The scoli are at the lateral longitudinal strip (scale bar = 50 µm). (**F**) The spiracle is shown in segment A5. (**G**) Fluorescence of the hair warts shows in segment A9 (scale bar = 50 µm).

T1 is the first thoracic segment, consisting of a prothoracic plate with a central groove that divides it into two parts. There are two scoli on each side, which have long white hairs surrounded by spine-like setae that point towards the head (Figure 5B,C,E). On each side, the scoli positioned at the subspiracular location (Sc3) have three spine-like setae and seven long white hairs (Figure 5F and Figure 6A,B). The scoli positioned at the subventral area (Sc4) above the first thoracic leg have five extending white hairs (Figure 5F and Figure 6A,B,H). The lateral side of T1 has a black background, adorned with yellow hair warts (Figure 6A and Figure 7A).

T2 and T3 both have a black background with a central band of red scoli, encircled by yellow hair warts (Figure 2, Figure 5C and Figure 7A). Each segment has six pairs of scoli in position: the scoli at the dorsal area (Sc1), scoli at the subdorsal area (Sc2), and scoli at the subspiracular area (Sc3) on the longitudinal stripe (Figure 6A,B,D–H).

Details about the scoli of thorax segments are as follows: Sc1: 923 µm diameter, 1 central white hair, 10–11 brown spine-like setae; Sc2: 640 µm diameter, 1 central white hair, 6 brown spine-like setae; Sc3: 350 µm diameter, 6 central white hairs, 3 spine-like setae on one side; Sc4: 150 µm diameter, 2–3 white hairs extending towards the abdomen (Figure 6A,B).

#### 3.1.4. Abdominal Segments

**Figure 8:**
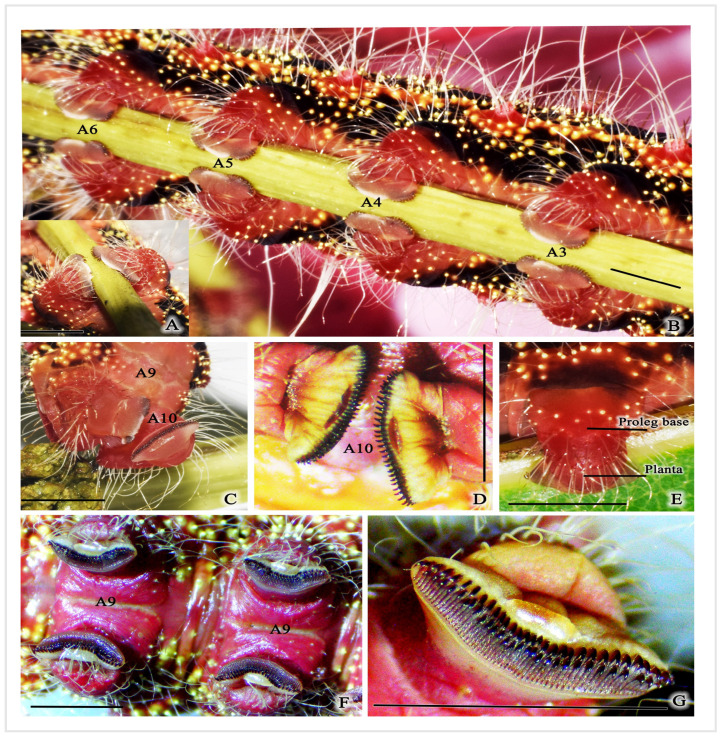
Component of the abdominal part of *C. trifenestrata* Helfer larva. (**A**,**B**) Prolegs of segments A3–A6. (**C**) Anal prolegs, prolegs on the last abdominal segment (A10). (**D**) Crochets, small hook-like structures. (**E**) Proleg and planta. (**F**) Four prolegs with crochets. (**G**) Enlarged image of crochets, two rows of small black hooks (biordinal arrangement) (scale bar (**A**–**G**) = 3 mm).

The abdomen of the larva comprises ten segments, each designated by a letter (A1–A10) (Figure 1 and Figure 7A). Segments A3–A6 have prolegs, which are fleshy and stub-like structures (Figure 7A and Figure 8A,B,E). Segment A10 has a pair of anal prolegs (Figure 8C,D). These prolegs have a pattern of small hook-like structures called crochets arranged in two rows, with biordinal arrangements (Figure 8F,G). Segments A1–A8 have a pair of spiracles on either side (Figure 7B,F). Segment A10 has an anal plate (Figure 7C).

Each segment has a black background, with a red–orange stripe in the middle, known as scoli (Figure 2 and Figure 7A,B). There are six pairs of scoli on each segment A1–A9, positioned as follows: the dorsal scoli (Sc1), the subdorsal scoli (Sc2), and the subspiracular scoli (Sc3) along the longitudinal stripe. Sc1 has 6–8 spine-like setae and one long white hair (Figure 7D). Sc2 has 3–6 spine-like setae with one long white hair. Sc3 has three spine-like setae and 3–5 long white hairs extending from the body (Figure 7B,D,E). Additionally, each segment has small yellow hair warts.

#### 3.1.5. Color and Pattern of the Whole Body

The fifth instar larva of *C. trifenestrata* Helfer display a range of colors visible to the human eye, including crimson–red, red, black, brown, yellow, pink, and white. These colors are arranged in different patterns that can be classified as bands of alternating transverse bands against a black background, red scoli and yellow hair warts, and a lateral longitudinal red stripe (known as the subspiracular line) with yellow hair warts (found in T2–T3, A1–A9 segments) (Figure 2 and Figure 7A).

The yellow hair warts on the thorax and abdomen of the larvae exhibit a fascinating feature when captured through flash-based DSLR cameras or conventional light microscopes. They display a luminous (glowing) appearance that is quite striking to the naked eye, as seen in Figure 6A and Figure 7A. Interestingly, even in defrosted larvae, the glow of these hair warts is still visible, albeit to a lesser extent. Observing Figure 7E–G, it is evident that there is a white glow at the tip of the yellow hair warts.

**Figure 9:**
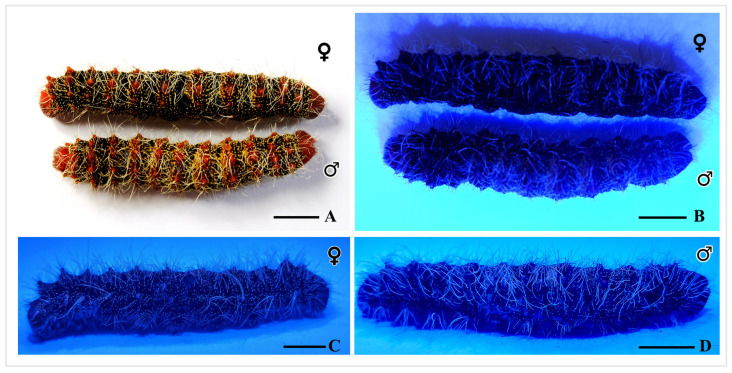
Fluorescence of male and female of fifth instar larva of *C. trifenestrata* Helfer. (**A**) The larva under white light. (**B**–**D**) The larva under UV light (scale bar (**A**–**D**) = 1 cm).

When exposed to LED UV light, fluorescence originates from hair warts, individual hairs, scoli, and hairs on the scoli of the cryopreserved *C. trifenestrata* Helfer larva (Figure 9B,C,D). The researchers’ observations suggest that the fluorescence is more pronounced and noticeable on the small hair warts and individual hairs on scoli than on the scoli themselves, which lack fluorescence in their spine-like setae.

### 3.2. Sexual Dimorphism in the Fifth Instar Larval Stage

**Figure 10:**
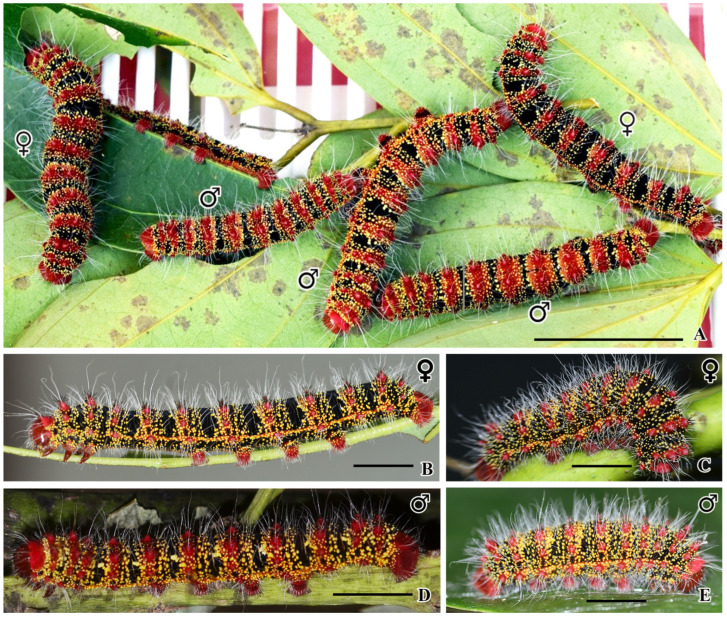
Live male and female larvae of *C. trifenestrata* Helfer. (**A**) A group of larvae feeding on cinnamon leaves. The females are larger and have a black background, while the males have smaller, more orange–crimson stripes on their wider scoli (scale bar = 3 cm). (**B**,**D**) Fifth instar larvae of both sexes. (**C**,**E**) Mature larvae shrinking before turning into pupae. Note that the glowing yellow hair bases are more visible in males (scale bar (**B**–**E**) = 1 cm).

**Figure 11:**
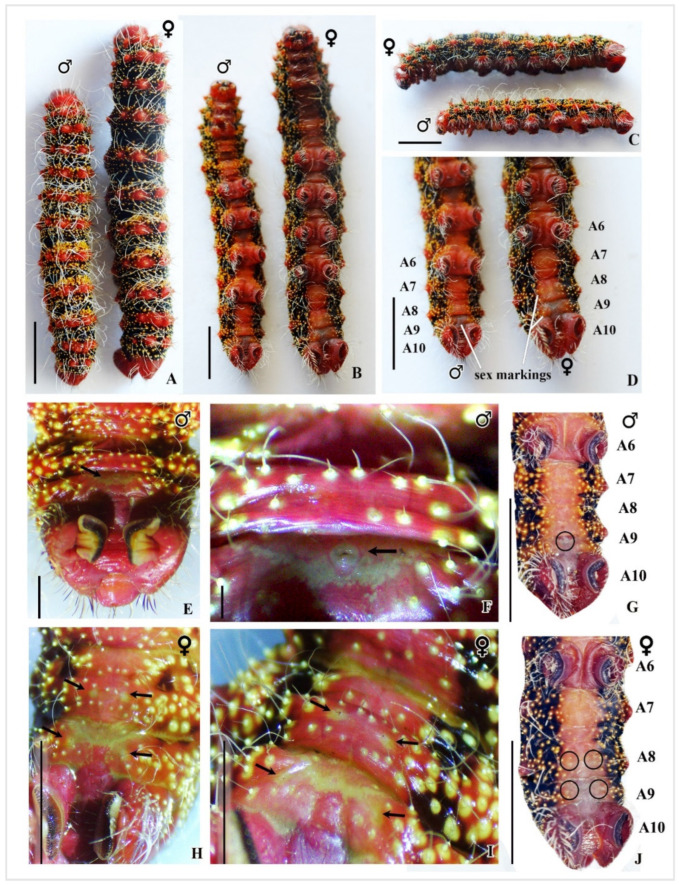
Dead specimen of *C. trifenestrata* Helfer male and female larva. (**A**) Dorsal view of the male and female. (**B**) Ventral view of the male and female. (**C**) Lateral view of the male and female. (**D**) Sex markings on ventral side of both sexes. (**E**–**G**) Male sex marking: One with a small gland between the eighth and ninth segments, indicated by arrows in (**E**,**F**), and encircled in (**G**). (**H**–**J**) Female sex marking: Two pairs of markings on the eighth and ninth abdominal segments, indicated by arrows in (**H**,**I**), and highlighted with four circles in (**J**) (scale bar (**A**–**J**) = 1 cm).

During the fifth instar larval stage, it is possible to distinguish between male and female larvae by examining their external characteristics. Female larvae have a longer body length of 6.8–7.3 cm, with narrow red–pinkish stripes, a clear black stripe, and two pairs of markings on the eighth and ninth abdominal segments. Conversely, male larvae have a shorter body length of 6–6.5 cm, wider red–pinkish stripes, one small gland located between the eighth and ninth segments, and a more significant number of glowing yellow warts on their thorax–abdominal segments. Figure 10A–E and Figure 11A–J provide visual representations of the differences between the two sexes.

The results presented in Figure 9 indicate the differences between male and female larvae that were exposed to LED UV light, in comparison with those exposed to only white light. When the fluorescence was compared, males were brighter than females. This is likely due to the fact that male larvae have a greater number of yellow hair warts and more hair than females, as can be seen in Figure 9A and Figure 11A.

## 4. Discussion

This study thoroughly investigates the external morphology of the fifth instar larvae of the Cricula wild silkmoth, *C. trifenestrata* Helfer, collected from a Chaiyaphum Province orchard in Thailand. It contributes significant insights into the larval development of this species.

### 4.1. External Morphology of the Larval Body

The morphological structure of the fifth instar larva of *C. trifenestrata* Helfer in its head, thorax, and abdomen closely resembles the typical structure of caterpillars belonging to the order Lepidoptera. Examples are the presence of an inverted Y-shaped suture, an adfrontal suture located on the front of the head, six stemmata on the head capsule, silk glands situated on the labium, and prolegs located on specific abdominal segments, such as A3, A4, A5, A6, and A10, and crochets (hooks), as documented by Scoble (1995) [16], Wagner and Hoyt (2022) [17], and Miller and Hammond (2003) [12].

Researchers have presented a comprehensive summary of key findings regarding the distinct morphological features of the fifth instar larvae of *C. trifenestrata* Helfer in comparison with other larvae of the Saturniidae family ([18]; Barrett and Kroening, 2003 [19]; and Liu, 2023 [20]). Notably, the larvae exhibit a yellowish-white, kidney-shaped clypeus contrasting with the crimson–red hue of the head capsule. The thoracic region displays a red thoracic shield in T1, with four scoli protrusions on the anterior plate edge adorned with spine-like setae, including a single long hair. Scoli distribution varies, with Sc3 along the lateral longitudinal line and Sc4 in the subventral area. Fluorescent yellow hair warts are observed in the lateral area of T1. Each T2–T3 segment showcases scoli (Sc1, Sc2, Sc3, and Sc4) with variations in size, the number of spine-like setae, and hairs. Abdominal segments (A1–A9) exhibit specific scoli with positional differences and alternating black and red–pink scoli strips, accompanied by fluorescent yellow hair warts. A distinctive red anal plate characterizes A10. These unique morphological features distinguish *C. trifenestrata* Helfer larvae from other lepidopteran caterpillars. Moreover, our study provides more descriptions of the fifth instar larva than in the reports of Hampson (1892) [6], Rono (2008) [7], Tikader et al. (2014) [1], and Magnussen et al. (2023) [3].

Morphological features such as scoli, hair warts, and aposematic coloration are often associated with defense mechanisms in Lepidoptera. However, direct evidence linking these structures to specific protective functions against predators or parasitoids remains limited. Fifth instar *C. trifenestrata* Helfer larvae exhibit distinct traits, including aposematic coloration, fluorescent hair warts, and pronounced scoli. While these features are hypothesized to play a role in deterring predators, definitive experimental validation is necessary. The following discussion examines these structures’ potential defensive roles based on the existing literature and highlights areas requiring further investigation.

### 4.2. Aposematic Coloration

The striking coloration of *C. trifenestrata* Helfer larvae observed in this study suggests that their vivid patterns may function as an essential component of their defense mechanisms. This hypothesis aligns with numerous studies indicating that noxious organisms frequently utilize aposematic or warning coloration to signal unpalatability to predators. For example, Wang et al. (2021) demonstrated that many caterpillar species exhibit bright colors to deter predation by signaling toxicity or unpalatability [21]. The distinctive pattern of the fifth instar *C. trifenestrata* Helfer larva includes alternating transverse bands of black and red scoli and fluorescent yellow hair warts, which may enhance the larva’s visibility and serve as a warning signal to predators. The longitudinal red stripe running along the larva’s body, adorned with additional fluorescent yellow hair warts, may amplify its conspicuousness.

A novel finding from this study is the luminescence of the yellow hair warts in *C. trifenestrata* Helfer larvae under both white and UV light. These luminous structures, particularly visible in flash-based DSLR camera images and light microscopy, produce a striking glow on the thorax and abdomen. Remarkably, this luminescence persists in defrosted larvae, and under UV light, blue fluorescence emanates from the base of the hair warts, individual hairs, and scoli. These observations suggest a unique bioluminescent property that may contribute to the larva’s defensive arsenal, although the underlying mechanisms warrant further exploration.

The fifth instar *C. trifenestrata* Helfer larva provides a compelling example of morphological adaptation through aposematic coloration. Its luminescent yellow hair warts likely enhance its deterrent capabilities against predators. However, the defensive adaptations observed in *Cricula* larvae appear to be species-specific. Comparative studies reveal significant differences in coloration among related species, such as *C. andamanica* and *C. andrei*. For instance, the fifth instar *C. andrei* larva exhibits a green body with light yellow scoli and black spine-like setae [18]. These interspecific variations highlight the influence of genetic and environmental factors in shaping the external characteristics of these larvae.

Despite the hypothesized protective functions of *C. trifenestrata* Helfer’s coloration and structural features, additional research is required to substantiate these claims. For example, Ruiz-García (2020) reported that larvae with warning coloration did not universally deter natural enemies. This finding underscores the possibility that the defensive efficacy of aposematic coloration may vary depending on the predator species, suggesting that its role in deterring predation could be context-dependent [22]. To address these gaps, behavioral assays or predator-preference tests involving natural predators are necessary to assess whether these morphological features effectively reduce predation risk. Additionally, experimental manipulation, such as modifying or masking specific coloration patterns, could provide critical insights into their functional roles in predator deterrence.

### 4.3. Scoli

In this study, the authors examined the fifth instar larva of *C. trifenestrata* Helfer, which displays prominent scoli on each thoracic and abdominal segment. These scoli are organized in distinct bands extending from the dorsal to the ventral sides of the segments. Notably, the dorsal (Sc1) and subdorsal (Sc2) scoli exhibit a higher density of spine-like setae compared to the subspiracular (Sc3) and subventral (Sc4) scoli. This variation in setal density may serve as a defensive mechanism, particularly in the dorsal region, which is more exposed to predation. The dense spines on the thoracic segments may protect vital organs near the head, while forward-pointing spines likely deter predators from accessing the delicate intersegmental joints between the head and thorax.

The scoli of *C. trifenestrata* Helfer larvae may serve as visual and physical deterrents, warning potential predators of their toxicity or unpalatability, as observed in other Lepidoptera species [9]. Additionally, the presence of these spiny outgrowths could act as a mechanical defense, preventing predators from handling or consuming the larvae, a strategy widely observed among Saturniidae such as reported by Murphy et al. (2010) [23]. Miller and Hammond (2003) also suggested that physical adaptations such as spines, hairs, and bristles can irritate predators or cause discomfort upon contact, effectively reducing predation [12].

Several studies provide compelling evidence linking similar morphological structures to defense mechanisms in other Lepidoptera species. For instance, Sathe et al. (2015) reported that larvae of *Atada velutina* (Limacodidae) with branched spines exude formic acid, which is highly irritating to predators. Similarly, *Thosea cana* and *Parasa lepida* larvae produce formic acid from their spines, causing dermatitis upon contact [24]. The spines of *C. trifenestrata* Helfer, although their toxin remains unidentified, are hypothesized to serve a similar role, potentially acting as conduits for defensive chemicals. Sathe et al. (2015) also noted that spines of this species may cause severe effects resembling leprosy in humans, highlighting the need to explore the toxic properties of these structures further [24].

*C. trifenestrata* Helfer shares similar adaptations with other Saturniidae, where scoli are often brightly colored or uniquely structured to enhance their visibility and potential for deterrence. Nässig (1989) classified the scoli of *Cricula* larvae as “Point-bristly scolus”, characterized by stinging bristles that may contain venom [25]. Similarly, Deml and Dettner (2002) identified “Point-bristly scolus, type I” structures in *Cricula andrei* larvae, which contained liquid venom for defensive purposes [9]. Comparisons with these studies suggest that the scoli of *C. trifenestrata* Helfer likely belong to a similar classification and function as effective defensive adaptations.

Further research is essential to elucidate the detailed morphology, histology, and chemical composition of the spines of *C. trifenestrata* Helfer. A deeper understanding of the toxins they produce and their mode of action will clarify the precise role of these structures in predator deterrence and larval survival, as previously highlighted by Sathe et al. (2015) [24]. Such studies will also provide insights into the evolutionary significance of scoli in enhancing larval defense within the Saturniidae family.

### 4.4. Hairs

The larvae of *C. trifenestrata* Helfer are classified as hairy caterpillars due to their dense covering of hair distributed across the body. These hairs originate from two primary sources: the yellow hair warts and the scoli (Sc1–Sc4). Small yellow hair warts are scattered across the thoracic and abdominal segments, resulting in the spread of hair over the entire body. The hairs of *C. trifenestrata* Helfer may serve a defensive role, protecting the larvae from natural enemies such as predators and parasitoids.

Several studies provide evidence for the defensive functions of caterpillar hairs. Kageyama and Sugiura (2016) demonstrated that hairs act as physical barriers against parasitoids, inhibiting their ability to oviposit on the host. Long, thick hairs, in particular, provide an effective defense by physically preventing parasitoids from penetrating the caterpillar’s skin with their ovipositors [26]. Field observations by Magnussen et al. (2023) revealed that parasitoids tend to lay eggs in areas of the larval body that are less densely covered with hair, further emphasizing the protective role of these structures [3]. Wagner and Hoyt (2022) also reported that many caterpillars and moths utilize adaptive mechanisms, including physical defenses such as setae, scoli, and hairs, to escape predation and parasitoid attacks. Dense coverings of hair or setae function as formidable physical barriers, making it difficult for parasitoids to reach the larval surface to lay eggs. Additionally, some hairs contain irritating chemicals, further deterring predators and parasitoids [19].

In addition to their defensive role, hairs of *C. trifenestrata* Helfer larvae exhibit fascinating properties under UV light. Observations in this study revealed that certain hairs emit fluorescence, which may suggest a link to aposematic coloration. This phenomenon, wherein bright or unique coloration warns predators of unpalatability or toxicity, is widely observed in other Lepidoptera species. However, further research is required to understand the exact function of these glowing hairs in *C. trifenestrata* Helfer.

Future studies should focus on exploring the chemical composition of substances produced by the yellow hair warts and the mechanisms behind hair fluorescence. Investigating the potential role of fluorescent hairs in predator deterrence or communication could provide valuable insights into the evolution of defensive strategies in Saturniidae larvae. This line of research offers a promising avenue for uncovering novel adaptive traits in *C. trifenestrata* Helfer.

### 4.5. Sexual Dimorphism

Sexual dimorphism refers to physical differences between individuals of the same species that are not directly related to their reproductive functions, such as size, weight, coloration, markings, or behavioral and cognitive traits [8]. Morphological differences between males and females are forms of widespread intraspecific variation in Lepidoptera [27].

The fifth instar larva of *C. trifenestrata* Helfer presents sexual dimorphism. Female specimens of this species are characterized by a larger body size and slenderer scoli strips, along with darker black areas. In contrast, males tend to be smaller in size, with wider scoli strips, brighter orange–red bodies, and a greater number of fluorescent hair warts on the abdomen when compared to females. These color variations become increasingly pronounced as the larva advances to the pupal stage. Microscopic analysis showed that female larvae display two specific cream-colored spots on the eighth and ninth abdominal segments, known as Ishiwata’s fore and hind glands; while a creamy white spot, known as Herold glands, is found between the same segments in males. Additionally, the seventh to ninth segments in female larvae are longer and wider compared to males. Furthermore, under UV light, males exhibit more fluorescence, particularly on the abdominal segments, where more yellow hair warts are present than in females.

Sexual differences in the fifth instar larva are linked to size, sexual markings, color patterns, and fluorescence under UV light. Vollrath and Parker (1992) noted that sexual dimorphism has evolved across the animal kingdom, with some species having females that are larger than males (such as certain insects and spiders) [28], and others having males that are larger than females (such as male seals).

Male *C. trifenestrata* Helfer larvae exhibit brighter colors with a higher density of yellow hair warts on their dorsal surfaces compared to female larvae. In contrast, Liu (2023) found that female *Antheraea compta* Rothschild larvae display a more yellowish hue than males. Furthermore, the shiny scoli of female larvae tend to be purple-red, whereas those of males appear more purple-blue [20]. These color and luminescence variations are believed to be linked to aposematic coloration, which functions as a warning signal rather than a signal for mate selection, as the larvae are not in a reproductive stage. Further investigation is required to understand why male *C. trifenestrata* Helfer larvae possess more yellow hairs and exhibit greater bioluminescence than females. A comprehensive study of pigmentation in both sexes during both the larval and moth stages would provide critical insights into whether the differences in color patterns observed in larvae also influence mate choice during the moth stage.

**Figure 12:**
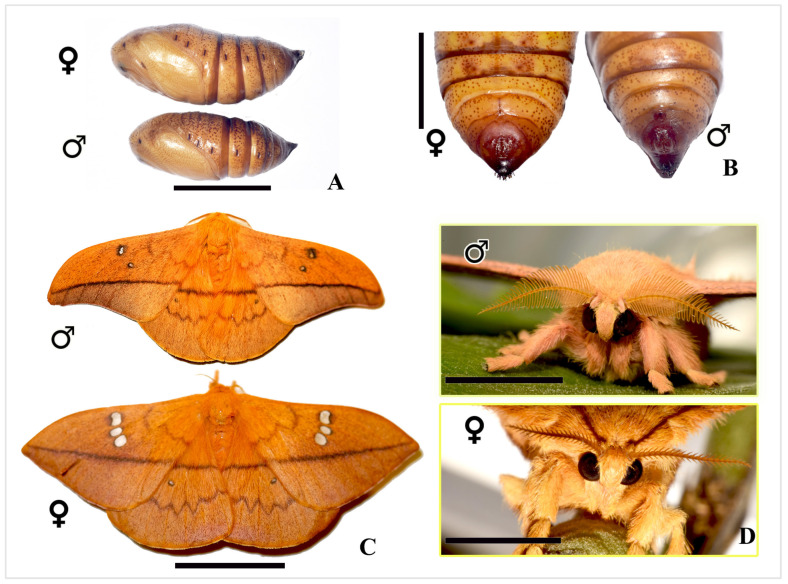
Sexual dimorphism in the pupal and adult stages of *C. trifenestrata* Helfer. (**A**) Lateral views of male and female pupae (scale bar = 1 cm). (**B**) Ventral views of the pupae, showing the fine longitudinal line on the eighth abdominal segment of females, which is absent in males (scale bar = 0.5 cm). (**C**) Dorsal views of male and female adult moths (scale bar = 2 cm). (**D**) Male moths possess quadripectinate antennae, while females have bipectinate antennae (scale bar = 5 mm).

While the primary focus of this study is the sexual dimorphism observed in the fifth instar larval stage of *C. trifenestrata* Helfer, sexual dimorphism extends into the pupal and adult stages. In the pupal stage, males and females exhibit significant differences in size and shape, with males being smaller and more streamlined, while females are larger and more robust (Figure 12A). Female pupae also display a fine longitudinal line on the eighth abdominal segment, which is absent in males (Figure 12B). Sexual dimorphism is even more pronounced in the adult stage. Female moths are larger than males (Figure 12C) and possess bipectinate antennae, while males exhibit quadripectinate antennae (Figure 12D). The sexes also differ in coloration, with females displaying darker brown and orange scales, while males exhibit lighter golden-brown tones. Wing patterns also vary; females have three transparent spots on their forewings, while males exhibit one transparent and one dark spot (Figure 12C). Male moths generally have narrower wings with more pronounced coloration patterns, whereas females are larger and possess broader wings. These findings are consistent with earlier reports by Magnussen et al. (2023), which documented similar sexual dimorphisms across the life stages of *C. trifenestrata* Helfer [3]. The consistent differences in size, shape, coloration, and wing patterns across developmental stages highlight the importance of considering multiple life stages when studying sexual dimorphism in this species.

Furthermore, these insights into sexual dimorphism have practical implications for agricultural practices, biological research, and taxonomic studies. This study contributes to addressing gaps in earlier research, such as those identified by Tikader et al. (2014) [1] and Magnussen et al. (2023) [3], by providing a more comprehensive understanding of gender-specific traits in *C. trifenestrata* Helfer. By broadening the scope to include multiple life stages, this study underscores the progressive manifestation of sexual dimorphism and provides valuable insights into the biological and ecological significance of these differences in *C. trifenestrata* Helfer.

## 5. Conclusions

In this study, the authors examined the external morphology of the fifth instar larvae of *C. trifenestrata* Helfer from Chaiyaphum Province, Thailand, enhancing the understanding of their larval development and morphological adaptations. Key features include typical Lepidoptera traits, such as an inverted Y-shaped suture, adfrontal suture, six stemmata, and prolegs with crochets. Additional distinct characteristics are a yellowish–white, kidney-shaped clypeus; crimson–red head capsule; red thoracic shield with scoli protrusions; and alternating black and red–pink scoli strips on the abdomen. These morphological traits suggest potential defensive functions, including aposematic coloration, fluorescence yellow hair warts, and point-bristly scoli, which may deter predators or parasitoids. However, these hypotheses are based on observed morphology and indirect evidence, and further research is necessary to confirm their specific functions. The researchers also observed sexual dimorphism in the larvae, with females being larger and displaying darker scoli strips, while males have brighter colors and more fluorescent hair warts. This dimorphism extends to the pupal and adult stages, providing valuable insight into the species’ biology, with potential implications for pest management and taxonomy.

## Data Availability

The data supporting the findings of this study can be obtained from the corresponding author upon reasonable request.
